# Why Are We Distracted by Social Media? Distraction Situations and Strategies, Reasons for Distraction, and Individual Differences

**DOI:** 10.3389/fpsyg.2021.711416

**Published:** 2021-12-02

**Authors:** Christina Koessmeier, Oliver B. Büttner

**Affiliations:** Economic and Consumer Psychology, Department of Computer Science and Applied Cognitive Science, University of Duisburg-Essen, Duisburg, Germany

**Keywords:** social media, distraction, situations, strategies, individual differences, fear of missing out, self-control

## Abstract

Social media is a major source of distraction and thus can hinder users from successfully fulfilling certain tasks by tempting them to use social media instead. However, an understanding of why users get distracted by social media is still lacking. We examine the phenomenon of social media distraction by identifying reasons for, situations of, and strategies against social media distraction. The method adopted is a quantitative online survey (*N* = 329) with a demographically diverse sample. The results reveal two reasons for social media distraction: social (e.g., staying connected and being available) and task-related distraction (e.g., not wanting to pursue a task). We find individual differences in these reasons for distraction. For social distraction, affiliation motive and fear of missing out (FoMO) are significant predictors, while for task-related distraction, self-regulatory capabilities (self-control, problematic social media use) and FoMO are significant predictors. Additionally, typical distraction situations are non-interactive situations (e.g., watching movies, facing unpleasant tasks). Strategies used to reduce distractions mostly involved reducing external distractions (e.g., silencing the device). This paper contributes to the understanding of social media use by revealing insights into social media distraction from the user perspective.

## Introduction

Internet and smartphones enable users to be permanently online and permanently connected (Vorderer et al., 2018). As a consequence, users can permanently be distracted by social media. Social media distraction refers to the process by which social media cues draw individuals’ attention away from a task that they originally pursued (e.g., working). Due to especially mobile access to social media, distractions by social media can occur frequently. Previous studies on multitasking have consistently demonstrated negative effects of distraction on performance (Jeong and Hwang, 2016), on academic performance among students (Junco and Cotten, 2012; Giunchiglia et al., 2018) and on well-being (e.g., Brooks, 2015). By drawing away users’ attention, distractions take up limited cognitive resources.

Given these negative consequences, it is important to understand users’ underlying reasons for social media distraction. Understanding the reasons for social media distraction can help to increase users’ agency to deal with unwanted social media distractions. Therefore, our major goal is to identify the reasons that underlie users’ distraction by social media. Furthermore, we examine how these reasons for distraction relate to individual differences in general (e.g., self-control) and social media-specific traits (e.g., problematic social media use). Additionally, to fully understand the phenomenon of social media distraction, we identify typical situations in which users are distracted, and we examine which strategies people use to handle social media distractions.

## The Problem of Social Media Distractions

Because of human’s limited capacity to process information (Pashler, 1994; Lang, 2000), distraction is problematic. Thus, in order to fulfill specific tasks successfully, social media distractions should be minimized. *Distractions* are caused by task-irrelevant *stimuli* that interrupt goal-directed behavior (Clapp and Gazzaley, 2012). Such distractions should be ignored when people want to focus on a task that requires their undivided *attention* to fulfill a certain goal. For instance, when writing a paper or talking to someone, social media cues–as the irrelevant distractors in that situation–are distracting by drawing the attention away from the primary task.

We refer to *social media distraction* as the phenomenon of social media cues (the distractors) drawing the attention away from the task at hand and directing it instead toward social media. These cues can be external or internal (Wilmer et al., 2017). For instance, social media distraction can be external (i.e., from the environment), such as receiving a notification, or internal (i.e., from within a person), for example when a user starts thinking about social media (e.g., unanswered messages). While users are engaged in a task, mind wandering may lead to internal distraction (McVay and Kane, 2010). For instance, prior work showed that students’ mind frequently wandered to social media when learning online (Hollis and Was, 2016). Mind wandering has been attributed to failed attentional control (McVay and Kane, 2010).

When faced with internal or external social media distractions, users can determine how to react and handle the distraction. There are three possible reactions to social media distractions: (a) *ignoring* the distraction and going on with the task; (b) *stopping* the task to use social media instead; or (c) starting to *multitask* (frequent switching between the task and social media). Social media cues distract from a task and offer the option of using social media. Starting to use social media as a reaction to distraction can have various reasons and how users handle this distraction can differ. The consequence of distraction can be that users start using social media (b or c). One explanation for why users engage in social media instead of ignoring it when engaged in a task, may be a failure of self-control. Research has found that social media self-control failure is related to high social media use (Du et al., 2018, p. 68). Moreover, users may engage in social media use after getting distracted in order to procrastinate. Research has indicated that procrastination–“voluntarily delay an intended course of action despite expecting to be worse off for the delay” (Steel, 2007, p. 66)–is related to high social media use (Reinecke et al., 2018; Rozgonjuk et al., 2018). The distraction may offer users an option to procrastinate instead of working on their tasks. Concluding, a user’s reaction to distractions may be influenced by, for instance, a failure to control one’s social media use or by the desire to procrastinate.

### Situations Prone to and Strategies Against Social Media Distractions

Prior studies have usually focused on social media as a distraction in one specific situation. For instance, a review found that students frequently use social media while in a lecture, reading, or studying (Chen and Yan, 2016). Additionally, prior research has examined distractions while working (Brooks and Califf, 2017) or while actively participating in traffic (Gliklich et al., 2016). Moreover, previous research has investigated such effects in social situations, such as relationship formation (Przybylski and Weinstein, 2013) or romantic relationships (Roberts and David, 2016). To summarize, most previous studies have focused on one specific situation in which distraction is examined, but an overview of *typical distraction situations* is lacking in prior research. Therefore, the present study investigates which distraction situations are typical in users’ daily lives.

Different strategies may be needed to successfully handle social media distraction, but so far it is unclear which strategies individuals already use. For instance, previous research has argued that closing social media tabs in the browser, turning off notifications, and trying to put the device out of sight might reduce distractions (Carrier et al., 2015; Kushlev et al., 2016). To empower social media users to avoid distractions, it is first necessary to understand the strategies that people use. Therefore, we investigate which *strategies* are used most frequently. In summary, we seek to identify distraction situations and strategies (RQ1).

### Reasons for Social Media Distraction

Social media’s strong pull factor–others have described it as “hedonic appeal” (Brooks, 2015, p. 26) or temptation (Hofmann et al., 2017)–makes users “drawn to distraction” (Aagaard, 2015, p. 93) and leads them to override their primary goals and tasks. This strong pull of social media has a high potential for distraction. For instance, research has found that students cannot focus for long on a task such as studying, and that, on average, they switch to social media after about six minutes of focused work (Rosen et al., 2013) and react to notifications shortly after their arrival (Pielot et al., 2014). In order to limit these distractions so that goals can be successfully accomplished, it is necessary to understand the underlying reasons for the distractions.

According to the uses and gratifications (U&G) approach (Katz et al., 1974), users actively seek media to fulfill certain needs and gratifications. From a variety of media choices, users select those that they expect to fulfill their needs. Social and psychological factors as well as the context influence media use and effects (Rubin, 2002). Accordingly, we argue that social media distraction represents a user’s *active* choice to fulfill certain needs. Even though external distractions can occur uncontrolled, it is a user’s active choice how to handle these distractions. Similarly, users working on a task might be “hijacked by task-unrelated thought[s]” that may distract internally (McVay and Kane, 2010, p. 324). Relatedly, prior work indicated that being preoccupied with the online world increased mind wandering (Johannes et al., 2018a). Even though these internal distractions represent an attentional control failure, users can still choose how to handle such a brief moment of uncontrolled attention (i.e., how to react to an uncontrolled thought about social media that arises), similarly to a user’s choice of how to handle external distractions. Users can choose whether to give in to the distraction (and start using social media) or to ignore the distraction.

In light of U&G, we propose that a user’s *susceptibility* to social media distractions in a specific moment represents need satisfaction (e.g., to find out whether someone texted), even though this might conflict with the user’s current goal-relevant task. Users’ momentary needs might influence how susceptible they are to distractions since users’ needs may influence attentional control. This may result in mind wandering (internal distractions) or, for instance, looking at the smartphone (external distractions). Hence, we argue that user’s needs may influence how susceptible users are to distractions. Moreover, U&G has been widely used in previous research to investigate why people use media (Ruggiero, 2000). Similarly, we want to investigate why users get distracted by social media–that is, we are interested in the reasons for social media distraction.

Previous research has identified several motivations for using social media *in general*, such as to communicate (Whiting and Williams, 2013), to stay in touch (Papacharissi and Mendelson, 2010), to feel connected to others (Quan-Haase and Young, 2010), to escape (Papacharissi and Mendelson, 2010), or to pass time (Whiting and Williams, 2013). These motivations for social media use describe the overall reasons for signing up to and using social media. The present research, by contrast, focuses on the *reasons for social media distraction*. We examine the underlying motivation for users’ increased susceptibility toward social media cues that draw the attention away from a task to which an individual originally attended. Motivations for social media use and reasons for distraction may overlap to a certain degree, but nevertheless reflect different aspects. For instance, most people do not sign up at a social media platform with the intention to procrastinate on their homework.

From a U&G perspective, it is relevant to examine the reasons for social media distraction. As Rubin (2002) argued, “to explain media effects, we must first understand the characteristics, motivations, selectivity, and involvement” (p. 526), because these can “have important implications for media effects” (p. 536). For example, research has found that users’ motivation influences which social media features they use (Smock et al., 2011). Therefore, understanding the reasons for social media distraction is a first step that enables future research to investigate the possible influences of distraction on different behaviors. Hence, this study investigates why users react to rather than ignore distracting social media cues when they are working on a task. Our second research question seeks to identify users’ reasons for distraction by social media (RQ2). In particular, we are exploring which different types of social media distraction exist.

### Individual Differences and Social Media Distraction

According to U&G, individual differences influence media use and effects (Rubin, 2002; Sherry and Boyan, 2008). Similarly, we expect that users’ traits contribute to individual differences in social media distraction. Based on the current literature, we identified a set of traits that we considered relevant for explaining why users are distracted by social media: basic motives, self-control, impulsivity, problematic social media use, and fear of missing out (FoMO). Our rationale was to include general traits, which are not exclusively related to social media, as well as traits that are specific to social media use. Given that we did not know in advance which factors of reasons for distraction by social media would emerge from the analysis, we could not formulate specific hypotheses regarding which of the trait variables correlated with which type of distraction. Therefore, we adopted an exploratory approach. Our third research question investigates how individual differences influence the reasons for social media distraction (RQ3).

We included *basic motives* to address general individual differences in motivations that underlie behavior. Motives refer to stable “predisposition[s] to approach a particular class of incentives… or to avoid a particular class of threats” (Trash et al., 2012, p. 141). Previous research has identified achievement, power, and affiliation/intimacy as basic motives (Emmons, 1997; Schönbrodt and Gerstenberg, 2012). Achievement refers to striving for adherence to excellence and mastering challenging tasks. Power describes the endeavor to impact others (regarding their attitudes or behaviors) and being concerned about status and prestige. Affiliation refers to the wish to have social relations, while intimacy refers to the motive of having strong social interactions and being close to others. For instance, research has shown that, of these motives, power and affiliation are related to a positive attitude toward social media (Sariyska et al., 2019). We included these explicit motives because they represent overarching motivations for users’ behavior.

*Self-control* and *impulsivity* are indicators of users’ self-regulatory abilities. Self-regulation–inhibiting or overriding impulses and temptations in order to achieve a higher-level goal (Baumeister and Heatherton, 1996)–is necessary for the ability to resist the temptation of social media distractions. Previous literature has discussed self-control as a predictor for media use (Reinecke and Hofmann, 2016; Hofmann et al., 2017), demonstrating that low self-control and high impulsivity relate to higher multitasking (Wang et al., 2012; Sanbonmatsu et al., 2013) or to a fast response to messages (Berger et al., 2018).

*Fear of missing out* refers to the FoMO on rewarding experiences others might have (Przybylski et al., 2013). Previous research has found that it is important for people to stay socially connected (Przybylski et al., 2013). In particular, FoMO is related to higher social media use (Przybylski et al., 2013), especially in situations when pursuing a task such as studying (Milyavskaya et al., 2018). Users might show an increased susceptibility to social media distraction to avoid the feeling of missing out.

*Problematic social media use* might also influence social media distraction. In its extreme form as social media addiction, it is characterized by a preoccupation with social media, loss of control and problems in social interactions (Wegmann et al., 2017), and low self-control (Wegmann et al., 2015). This suggests that users with a tendency toward social media addiction are also more susceptible to social media distraction.

### Overview of the Study

This study used an exploratory approach to address the three research questions. We explored social media distraction, in particular investigating in which situations people are most likely to be distracted and which strategies they use to regulate their distraction (RQ1). Second, we wanted to identify the reasons for social media distraction–that is, we investigated why people get distracted by social media (RQ2). Finally, we investigated if social media distraction depends on individual differences (RQ3) in trait variables (general motives, self-control, impulsivity) and social media-specific variables (FoMO, problematic social media use).

Our methodological approach consisted of two steps: First, in preliminary studies, we conducted qualitative interviews to uncover users’ reasons for distraction, distraction situations, and strategies, which we then pre-tested as items in follow-up studies. Second, for our main study and the focus of this paper, we conducted a quantitative online survey with a large and heterogeneous sample. Data and supplementary material are available via the Open Science Framework (OSF): https://osf.io/5pvj6/.

## Materials and Methods

### Preliminary Studies

The goal of the preliminary studies was to develop the items for the research focus (reasons for distraction, distraction situations, and strategies) used in the main study. In 15 semi-structured qualitative interviews (each with a duration of 15–20 min), we asked students questions relating to why they get distracted, in which situations this was most likely to happen, and what strategies they used to limit their distraction. We asked students since we assumed these are particularly prone to distraction. Five interviews each focused on one of the three topics (reasons for social media distraction, situations for social media distraction, strategies to reduce distraction). Questions started openly but included targeted questions to find out more about the three topics. Subsequently, we developed the items based on the insights gained from the interviews; namely, we extracted and aggregated the main reasons, situations, and strategies that interviewees pointed out and we refined these based on the literature. We developed these items without any potential underlying factors in mind. We then pre-tested and refined the items in two questionnaire studies (*N*_1_ = 92; *N*_2_ = 127) before including them in the main study to get a first impression of social media distractions and our items. This preliminary work resulted in the scales for reasons for distraction, distraction situations, and strategies used to limit distraction, which we then used and explored in the main study (see Measures section for a detailed description and Tables 3–5 for the items). Further information on the preliminary material is available online in our OSF repository.

### Main Study

The main study was a quantitative survey. To begin, the survey asked about social media use and social media distraction in general. The survey then focused on reasons for social media distraction, potential distraction situations, and strategies used to limit distractions. The survey looked into individual differences regarding FoMO, problematic social media use, explicit motives, self-control, and impulsivity. Lastly, the survey included socio-demographic variables. The study was approved by the department’s ethics committee.

#### Sample

For the survey, we recruited social media users via an online access panel in Germany. The prerequisites for participation in the study were having at least one social media account and using social media at least once per week. Since the aim of our study was to investigate social media distraction, it is necessary that only those people participate that are familiar with social media. In order to reach a sample reflecting a broad selection of social media users, we recruited participants aged between 18 and 69 years.^1^ Overall, 382 social media users from Germany participated in the study^2^. To ensure data quality, we excluded 53 participants from the analysis. Of those, 10 users were excluded for interrupting survey completion. We excluded the fastest and slowest 5% (40 respondents) to control for people not filling out the survey with attention. Three participants were excluded because of inappropriate responses to open questions. Table 1 summarizes the socio-demographic details of the participants in our final data set used for the analysis.

**TABLE 1:** Socio-demographic details on sample.

	*N*	% of Sample	*M* (SD)
Age			42.58 (14.75)
**Gender**			
Female	168	51	
Male	161	49	
**Education**			
Not graduated from school	3	0.9	
Lower secondary school graduate	25	7.6	
Secondary school certificate	63	19.1	
Traineeship	73	22.2	
Higher education entrance qualification	91	27.7	
Bachelor (University degree)	23	7.0	
Master (University degree)	44	13.4	
Other	7	2.1	
**Occupation**			
Student (School)	2	0.6	
Student (University)	33	10.0	
In traineeship	8	2.4	
Employee	164	49.8	
Self-employed	21	6.4	
Homemaker	17	5.2	
Unemployed	15	4.6	
Retired	54	16.4	
Other	15	4.6	
**Marital status**			
Single	91	27.7	
In a relationship	80	24.3	
Married	126	38.3	
Divorced	27	8.2	
Widowed	5	1.5	

*N = 329.*

#### Measures^3^

For descriptive purposes, we assessed frequency of *social media use* (a few times a day, daily, a few times a week, once a week, once a month or less), social media use in hours per day (“How many hours do you use social media in a regular day? I use social media for… hours per day”), and for which social media platforms participants held an account. We assessed different facets of *social media distraction* with three single items: (1) degree of distraction (1 = *not much distracted by social media*; 5 = *very much distracted*); (2) reactivity to distraction (1 = *typically directly respond to notifications*; 5 = *rather take some time to react to a notification*); and (3) typical source of distraction (1 = *internally*–thinking about social media-related content; 5 = *externally*–receiving notifications). *Evaluation of distraction* measured how far participants perceive their social media distraction as problematic (Table 2; five items, scaled 1–5, α = 0.91). *Notification settings* assessed whether participants receive all, some, or no notifications, or never stay logged in.

**TABLE 2:** Descriptive measures on distraction by social media.

	*M*	SD
Degree of distraction	2.50	1.20
Source of distraction	2.91	1.28
Reactivity to distraction	3.29	1.07
**Evaluation of distraction behavior**	3.07	1.03
Unproblematic (1)–very problematic (5)	2.94	1.21
Not stressful (1)–very stressful (5)	2.82	1.20
Not disturbing (1)–very disturbing (5)	3.09	1.23
Not much (1)–very much time-consuming (5)	3.45	1.15
Not critical (1)–very critical (5)	3.05	1.19
**Evaluation of strategy use**	3.76	1.04
Unhelpful (1)–helpful (5)	3.81	1.08
Ineffective (1)–effective (5)	3.76	1.11
Useless (1)–useful (5)	3.78	1.43
Unsuccessful (1)–successful (5)	3.71	1.10

*N = 329.*

To find typical *situations* of social media distraction, participants rated 10 situations based on how often they perceived social media distraction in these situations (Table 3), ranging from (1) *never/rarely* to (5) *very often*. This scale measured the extent to which people perceived themselves as being typically distracted in certain situations. Moreover, to assess *reasons for social media distraction* (Table 5), participants rated each of the 16 reasons by determining how much the reason related to their distraction behavior. Participants had to think back to the previously mentioned distraction situations, complete the sentence “I get distracted in these situations, because …,” and rate each listed reason on a five-point rating scale ranging from (1) *totally disagree* to (5) *totally agree*. In order to discover people’s most common *strategies* to limit social media distraction, the survey asked participants to indicate which strategies they already used to reduce social media distraction. Participants rated, from (1) *never* to (5) *always*, how often they would use each of these 15 strategies (Table 4). Additionally, participants rated their *evaluation of strategies* used (Table 2, four items, scaled 1–5, α = 0.96) to reduce distractions.

**TABLE 3:** Situations prone to social media distraction.

	*M*	SD
While watching a movie/series	2.85	1.30
When I do not want to start with my task after a break	2.47	1.24
When I want to delay the start of a task	2.22	1.21
When I have to do an unpleasant task	2.16	1.19
When I would have other, more important tasks to do	2.12	1.13
While studying/working	2.06	1.13
When talking to family/friends/others	1.86	1.02
When I am eating with others	1.64	0.92
When I actively participate in traffic	1.57	0.93
In a meeting	1.37	0.83

*N = 329, English translation of the original German items used in this study, original items are in our OSF repository. We gave a brief description of distraction by social media: it was described as referring to situations in which people, while performing a task, are distracted by social media, either internally or externally. Introduction to these items: “When you are in one of the following situations, how often do you get distracted?”.*

**TABLE 4:** Strategies used to reduce distractions by social media.

	*M*	SD
… silence my devices	3.51	1.45
… leave my device at a different location (e.g., other room, at home)	2.58	1.36
… deactivate notifications	2.55	1.48
… place the device out of reach	2.49	1.35
… turn my device around so that I cannot see any notifications	2.41	1.36
… turn off my device	2.33	1.36
… deactivate the Internet connection	2.25	1.35
… log off my social media accounts	2.22	1.41
… activate flight mode	2.10	1.30
… treat myself for successfully avoiding social media	1.64	1.03
… use apps/plug ins to control my social media use	1.59	1.06
… delete my social media apps (temporarily)	1.57	1.02
… lock my device away	1.55	1.00
… give my device to another person (e.g., spouse)	1.50	0.96
… delete my social media accounts	1.44	0.91

*N = 329; English translation of the original German items used in this study, original items are in our OSF repository. Introduction to these items: “In order to be less distracted by social media, I…”; Device refers to all that are used for accessing social media.*

**TABLE 5:** Exploratory factor analysis of reasons for distraction.

	*M*	SD	Factor loading	
			1	2
**Factor 1: social reasons**				
… It is important to me to directly reply	2.18	1.21	**0.86**	−0.14
… I always directly reply	2.21	1.23	**0.77**	−0.06
… I always want to be up to date	2.42	1.28	**0.77**	0.07
… I want to know what is happening	2.80	1.28	**0.74**	0.09
… I want to keep up with what others are doing	2.51	1.23	**0.73**	0.09
… I want to know what others are writing/posting/liking/sharing	2.72	1.30	**0.69**	0.06
… My friends expect me to react	2.46	1.23	**0.68**	0.02
… I want to stay in touch with friends	3.13	1.27	**0.65**	0.09
**Factor 2: task-related reasons**				
… I am not interested in pursuing my tasks	2.45	1.29	−0.10	**0.93**
… I want to escape an (unpleasant) situation	2.21	1.22	−0.03	**0.87**
… I want to get distracted	2.52	1.31	0.07	**0.71**
… I cannot concentrate	2.53	1.23	0.10	**0.67**
… I am bored	2.98	1.34	0.09	**0.63**
… I got interrupted by a notification while pursuing a task	2.34	1.22	0.21	**0.58**
… I am seeking entertainment and fun	2.86	1.28	0.29	**0.51**
*Eigenvalue*			4.82	3.91
*Explained Variance*			32%	26%

*N = 329, EFA with principal axis factoring and oblimin rotation; factor loadings >0.50 in bold; English translation of the original German items used in this study, original items are in our OSF repository; Introduction to these items: “I get distracted by social media in these situations, because…”.*

*Problematic social media use* was measured with the Internet addiction scale modified for social networking sites (s-IAT-SNS; Wegmann et al., 2015), with the two dimensions *loss of control* (six items, α = 0.89) and *craving* (six items, α = 0.91), rated on a five-point rating scale ranging from (1) *never* to (5) *very often*. *FoMO* (Przybylski et al., 2013) was measured with the revised FoMO scale (Wegmann et al., 2017), assessing *online* (seven items, α = 0.86) and *offline* (five items, α = 0.90) FoMO on a five-point rating scale ranging from (1) *totally disagree* to (5) *totally agree*.

We measured basic *explicit motives*–achievement (α = 0.88), power (α = 0.86), affiliation (α = 0.81), and intimacy (α = 0.80)–with the Unified Motivations Scale (UMS-6; Schönbrodt and Gerstenberg, 2012), using six items for each motive. We measured *self-control* using the German version of the Brief Self-Control Scale (Tangney et al., 2004; Bertrams and Dickhäuser, 2009; α = 0.84). We assessed *impulsivity* with the short form of the Barratt Impulsiveness Scale (BIS-15) in German (Meule et al., 2011; α = 0.81).

## Results

### Descriptive Summary of Social Media Use and Distraction

Participants estimated using social media on average 2.2 h per day (SD = 2.3). The most frequently used social media platform^4^ was WhatsApp (86%), followed by Facebook (82%), YouTube (67%), Facebook Messenger (49%), and Instagram (39%). Half of the participants (51%) reported having *some* notifications from social media activated, whereas 28% received *all* possible notifications, and 9% reported disabling all notifications.

Table 2 shows how participants perceived their distraction by social media. However, they reported that they experienced their distraction as rather negative. Participants stated that they generally took some time to respond to notifications instead of immediately reacting to them. The source of distraction seemed to be external rather than internal, that is, from notifications rather than from starting to think about social media.

### Situations and Strategies Against Social Media Distraction

First, we identified situations and strategies (RQ1). Situations typical for social media distraction are presented in Table 3. The situations in which people reported getting distracted most often were while watching movies/series, when trying to avoid returning back to a task, or when they wanted to delay the start of a task.

Strategies that were used to reduce distractions are presented in Table 4. The most common strategies were silencing the devices, leaving the devices somewhere else, or deactivating notifications. Overall, participants evaluated their use of strategies to limit social media distractions as moderately effective (*M* = 3.76, SD = 1.04).

### Reasons for Social Media Distraction

Moreover, we investigated the reasons for social media distraction (RQ2). Table 5 shows the descriptive statistics of the items measuring reasons for distraction. To find underlying types of reasons, we conducted an exploratory factor analysis (EFA). EFA is used when the underlying factor structure is not known, as it was the case in our study. We calculated the EFA using oblimin rotation^5^ and principal axis factoring.^6^ With regard to sampling adequacy, the Kaiser–Meyer–Olkin (KMO) measure showed acceptable results: overall, KMO = 0.94 and all individual KMO values were >0.87. Bartlett’s test of sphericity indicated that correlations were sufficiently large: χ^2^ (105) = 3259.72, *p* < 0.001. The EFA yielded two factors with eigenvalues greater than 1 and the scree plot indicated that two factors were suitable. Overall, the two factors explained 58% of the variance.

Table 5 shows the rotated factor loadings of the structure matrix.^7^ Factor loadings were >0.5; that is, they were well above the recommended threshold of >0.4 (Stevens, 2009; Field, 2013), and they showed no substantial cross-loadings on the other factor (<0.3; Stevens, 2009). Conceptually, Factor 1 relates to *task-related reasons* for distraction and indicates people being distracted by social media because they try to avoid tasks or do not want to do what they ought to, are bored, or cannot concentrate. Factor 2 relates to *social reasons* for distraction: people are distracted by social media because they want to feel connected, want to stay in touch, or feel the urge to reply. Hence, reasons for distraction comprised the two factors of social distraction and task-related distraction (each with eight items). The internal consistency of both factors was good (social: α = 0.91; task-related: α = 0.90). As expected, both factors significantly correlated with each other (*r* = 0.67, *p* < 0.001) which indicates that the two types of distractions are not independent from each other, but rather depict different facets of distraction. Overall, the scale assesses how strongly people are distracted by social media due to social and task-related reasons.

### Individual Differences as Predictors of Social Media Distraction

We investigated which individual differences predicted users’ social media distraction (RQ3). Table 6 shows that all trait variables correlated with social distraction and task-related distraction. In order to analyze which of the traits are most important for each type of distraction, we calculated two hierarchical regressions, one with social distraction and one with task-related distraction as dependent variable.

**TABLE 6:** Descriptive statistics and correlations for reasons for distraction and traits.

Variable	*M*	SD	1	2	3	4	5	6	7	8	9	10	11
1. Self-control	3.28	0.67	–										
2. Impulsivity	2.11	0.43	−0.61***	–									
**Fear of missing out**													
3. Offline	2.02	0.94	−0.46***	0.30***	–								
4. Online	2.00	0.88	−0.33***	0.29***	0.70***	–							
**Problematic social media use**													
5. Loss of control	2.22	0.87	−0.43***	0.36***	0.54***	0.63***	–						
6. Craving	1.85	0.85	−0.36***	0.37***	0.56***	0.63***	0.83***	–					
**Basic motives**													
7. Achievement	3.12	0.90	0.10	–0.10	0.21***	0.29***	0.19***	0.18***	–				
8. Power	2.51	0.90	0.01	0.00	0.34***	0.40***	0.28***	0.33***	0.60***	–			
9. Affiliation	3.09	0.78	0.09	–0.10	0.16**	0.22***	0.05	0.01	0.45***	0.27***	–		
10. Intimacy	3.60	0.78	–0.03	−0.11*	0.08	0.04	0.06	–0.02	0.34***	0.07	0.47***	–	
**Reasons for distraction**													
11. Social	2.55	0.99	−0.26***	0.18***	0.47***	0.68***	0.47***	0.45***	0.24***	0.27***	0.26***	0.14*	–
12. Task-related	2.21	0.89	−0.40***	0.33***	0.52***	0.57***	0.60***	0.49***	0.22***	0.21***	0.18***	0.15**	0.67***

N = 329. Pearson’s r correlation coefficient; *p < 0.05, **p < 0.01, ***p < 0.001.

In step 1 of the hierarchical regressions, we entered the general trait variables basic *motives* (achievement, power, affiliation, intimacy), *self-control*, and *impulsivity* as predictors. For *social distraction* (Table 7), these predictors accounted for about 20% of variance; power, affiliation, and self-control emerged as significant predictors. The stronger the power (β = 0.18) and affiliation (β = 0.21) motive, the higher was social distraction. Higher self-control (β = −0.25) reduced social distraction. For *task-related distraction* (Table 8), the model accounted for 25% of variance; achievement, self-control, and impulsivity emerged as significant predictors. The higher the achievement motive (β = 0.14) and impulsivity (β = 0.16), the higher was task-related distraction. Again, higher self-control (β = −0.32) was associated with reduced task-related distraction.

**TABLE 7:** Hierarchical regression examining the effect of traits on social distraction.

	*b*	SE	β	*p*	*R* ^2^	Δ *R*^2^	*F*	*p*
Step 1					0.197			
Constant	1.93	0.64		0.003				
**Basic motives**								
Achievement	0.07	0.08	0.07	0.350				
Power	0.20	0.07	0.18	0.005				
Affiliation	0.26	0.08	0.21	<0.001				
Intimacy	0.01	0.08	0.01	0.914				
Self-control	–0.37	0.09	–0.25	<0.001				
Impulsivity	0.13	0.15	0.06	0.361				
Step 2					0.488	0.291	45.32	<0.001
Constant	1.04	0.55		0.057				
**Basic motives**								
Achievement	–0.01	0.06	–0.01	0.902				
Power	–0.02	0.06	–0.02	0.750				
Affiliation	0.14	0.06	0.11	0.027				
Intimacy	0.08	0.06	0.06	0.208				
Self-control	–0.12	0.08	–0.08	0.142				
Impulsivity	–0.10	0.12	–0.04	0.418				
**Problematic social media use**								
Loss of control	0.04	0.09	0.04	0.634				
Craving	0.05	0.09	0.04	0.595				
**Fear of missing out**								
Offline	–0.07	0.06	–0.06	0.291				
Online	0.72	0.07	0.64	<0.001				

*N = 329.*

**TABLE 8:** Hierarchical regression examining the effect of traits on task-related distraction.

	*b*	SE	β	*p*	*R* ^2^	Δ *R*^2^	*F*	*p*
Step 1					0.252			
Constant	1.95	0.62		0.002				
**Basic motives**								
Achievement	0.15	0.07	0.14	0.036				
Power	0.11	0.07	0.10	0.116				
Affiliation	0.14	0.07	0.11	0.059				
Intimacy	0.07	0.07	0.05	0.368				
Self-control	–0.47	0.09	–0.32	<0.001				
Impulsivity	0.36	0.14	0.16	0.012				
Step 2					0.452	0.200	28.91	<0.001
Constant	0.49	0.56		0.388				
**Basic motives**								
Achievement	0.09	0.06	0.08	0.158				
Power	–0.06	0.06	–0.06	0.312				
Affiliation	0.07	0.07	0.05	0.309				
Intimacy	0.08	0.06	0.06	0.227				
Self-control	–0.13	0.09	–0.09	0.136				
Impulsivity	0.20	0.12	0.09	0.107				
**Problematic social media use**								
Loss of control	0.47	0.09	0.41	<0.001				
Craving	–0.19	0.09	–0.17	0.039				
**Fear of missing out**								
Offline	0.14	0.07	0.13	0.038				
Online	0.27	0.08	0.24	<0.001				

*N = 329.*

In step 2, we included the social media-specific variables *problematic social media use* (craving and loss of control) and *FoMO* (online and offline) as predictors. For *social distraction* (Table 7), these additional predictors increased the explained variance significantly to 49%. While affiliation was still a significant predictor (β = 0.11), power and self-control were no longer significant. In addition, online FoMO emerged as the strongest predictor of social distraction (β = 0.64). For *task-related distraction* (Table 8), the additional social media-specific predictors increased the explained variance significantly to 45%. None of the general trait variables remained significant; instead, all social media-specific variables significantly predicted task-related distraction. While loss of control (β = 0.41), offline FoMO (β = 0.13), and online FoMO (β = 0.24) were associated with higher task-related distraction, craving (β = −0.17) was associated with lower task-related distraction.

## Discussion

This research examined distraction by social media, and, more specifically, when, and why people are distracted, and what they do to reduce their distraction. We examined this with a larger German sample that is diverse in terms of demographic characteristics such as age, educational background, and occupation (see Table 1) and by investigating social media distraction in general, rather than focusing only on one social media platform. We identified typical distraction situations (e.g., when pursuing a task) and typical strategies users employ to be less distracted (e.g., silencing notifications). By focusing on reasons for distraction, we identified two types of social media distraction: social distraction and task-related distraction. These types of distraction differed in their association with individual differences in basic motives, self-regulatory abilities, problematic social media use, and FoMO.

### Situations of and Strategies Against Social Media Distraction

According to U&G, the environment influences media use and effects (Ruggiero, 2000; Rubin, 2002). Against this backdrop, we identified typical situations in which participants were distracted by social media (RQ1). This extends previous research, which has focused on social media distraction only in one particular context (e.g., studying; Chen and Yan, 2016). An interesting finding is that social media distraction occurs not only in non-interactive situations (e.g., when working on a task or watching a movie), but also when users are interacting with other people (e.g., when talking to others or when in a meeting). Although participants indicated that distraction in interactive situations is less frequent than in non-interactive situations, previous research has revealed that distraction in interactive situations may have strong negative effects, for instance, during social interaction it can negatively affect well-being (Xu et al., 2016) or relationship formation (Przybylski and Weinstein, 2013). Previous research has also argued that social media is used to escape unpleasant situations (Reinecke et al., 2018).

In addition, our study identified strategies that people use to reduce social media distraction. The most popular strategies, such as silencing the device, deactivating notifications, or placing the device out of sight, address external distractions. These strategies tackle the problem that push notifications demand users’ attention (Hofmann et al., 2017). Previous research suggests that such strategies are indeed successful. Simply being able to see the device is already distracting (Johannes et al., 2018b). Along similar lines, Aagaard (2015) found that students close their laptops strategically to reduce in-class distractions. However, strategies reducing external distractors (e.g., silencing notifications, relocating the device) may not suffice in reducing distractions (Pielot et al., 2014), because these strategies still allow easy access to social media, rely on internal control capabilities, and people may still be distracted internally (e.g., thinking about unread messages or likes). Participants also reported more drastic strategies, such as deleting accounts or apps. Obviously, our findings suggest that more drastic (and probably more effective) strategies are less likely to be adopted.

### Distraction Due to Social and Task-Related Reasons

By focusing on users’ underlying reasons (RQ2), we identified two types of social media distraction: social distraction and task-related distraction. *Social distraction* refers to an increased susceptibility to social media distractions because of striving for social connection and fulfilling others’ expectations. This corresponds to previous research arguing that social cravings motivate multitasking (Wang et al., 2012), problematic smartphone use (Seo et al., 2015), or distracting behavior (Clayson and Haley, 2013; Bayer et al., 2016), because social media use is socially rewarding (Bayer et al., 2016). Other studies have found that communicating with and being concerned about others are dominant reasons for in-class social media use (Clayson and Haley, 2013) and that social pressure is a main reason for quickly reacting to notifications (Pielot et al., 2014).

*Task-related distraction*, on the other hand, refers to an increased susceptibility to social media distractions in order to avoid unpleasant tasks, or to make uncomfortable situations more pleasant. This finding aligns with U&G research, which has often highlighted that people use media for entertainment or to avoid unpleasant thoughts (Ruggiero, 2000). Additionally, previous work has argued that people use (social) media to regulate their mood (Hofmann et al., 2017; Reinecke et al., 2018) or to make tasks more entertaining (Wang and Tchernev, 2012). For instance, previous research has suggested that students use social media during classes to procrastinate (Rozgonjuk et al., 2018) or out of boredom (Clayson and Haley, 2013). To summarize, the identified types of distraction indicate which possible gratifications make people more susceptible to social media distraction. From a U&G perspective, an investigation of these underlying reasons for social media distraction is important because, as Rubin (2002) argued, the motivations for media use influence the effects of media on its users.

### Individual Differences and Distraction

Our research aim was to examine whether individual differences in general and social media-specific traits in particular explain social media distraction (RQ3). We investigated various predictors that differ substantially for social versus task-related distraction. This underlines that social and task-related distraction are indeed different types of distraction because they are driven by different psychological processes. Thus, our results correspond to previous research on U&G stating that individual differences influence media use (Rubin, 2002; Sherry and Boyan, 2008).

When considering the general traits (hierarchical regression step 1), both types of distraction were predicted by lower self-control; in the case of task-related distraction, additionally by lower impulsivity. Importantly, social and task-related distraction differed in basic motives. Social distraction was predicted by strong affiliation and power motives. This indicates that social media distraction might be driven not only by the striving to connect with others but also by the exertion of power over others. For instance, previous research has argued that feeling socially excluded, in particular, makes users turn to social media (David and Roberts, 2017), indicating that a susceptibility to social media would be motivated by the need for social connection. Task-related distraction, by contrast, was predicted by a strong achievement motive. At first, this seems contrary to previous research arguing that people turn to media when faced with tasks that are “demanding, complex, unpleasant, boring or anxiety-inducing” (Reinecke et al., 2018, p. 864), and that students are susceptible to distractions in a difficult lecture (Aagaard, 2015). However, it fits well to research that has linked perfectionism as well as low self-control to procrastination (Ferrari, 1992; Przepiórka et al., 2019). Overall, the findings on the relationship between social media distraction and general traits provide two major insights. First, lower self-regulatory abilities contribute to social media distraction. This is in line with previous research that has conceptualized problematic social media use as a problem with self-control (Wegmann et al., 2015). In addition, it corresponds to the literature on mind wandering, identifying internal distractions as failed attentional control (McVay and Kane, 2010). Second, the findings show that, in addition to self-regulation, users’ motivational dispositions have additional explanatory power for social media distraction. This suggests that taking users’ motives into account, as suggested by U&G (Rubin, 2002), provides a more complete picture of social media distraction than the self-control perspective alone.

When including social media-specific variables (hierarchical regression step 2), the pattern of predictors changes, but substantial differences between social distraction and task-related distraction persist. For social distraction, the affiliation motive is still a significant predictor, but FoMO emerged as the most important predictor. This result is not surprising since FoMO refers to the striving to stay socially connected (Przybylski et al., 2013) and is related to higher social media use (Przybylski et al., 2013; Hunt et al., 2018). For task-related distraction, problematic social media use in the form of loss of control and craving are significant predictors. These refer to more social media-specific aspects of self-control and thus seem to replace the more general predictors–namely, self-control and impulsivity–identified in step 1. This corresponds to previous research showing that problematic social media use is associated with lower productivity (Duke and Montag, 2017). In addition, FoMO contributes to task-related distraction, which suggests that users neglect their tasks in favor of not missing out on things online as well as offline. Taken together, the findings show that users with low self-regulatory abilities and high FoMO are more prone to task-related distraction. For social distraction, FoMO is the most important predictor and users do not need to have low self-control to be susceptible to social media distractions.

### Limitations

Our study has certain limitations, which, at the same time, point to opportunities for future research. First, this study focused on users’ perception of distraction and thus is based on participants’ self-reports to capture their perception. In order to expand this perspective, future research should relate these subjective perceptions to more objective measures of social media distraction. For instance, use eye-tracking could be used to examine whether self-reported social media distraction goes along with higher visual distractibility by social media cues (see Serfas et al., 2016).

Second, we identified individuals’ use of strategies against distraction, but the effectiveness of these strategies remains unclear. By exploring users’ popular strategies, we tackled the call to investigate strategies that are realistically used in everyday situations (Chen and Yan, 2016). The next step should be to empirically test which of these strategies really help in reducing distractions. Furthermore, the popular strategies found here focus on reducing external distractions. Thus, future research could investigate strategies against internal distractions because previous research has indicated that reducing internal distractions might require different strategies (Rosen et al., 2013).

Third, we chose an exploratory approach. Hence, it is up to future research to explore causal relations. In our study, we identified the two reasons for distraction, but it is thus far unclear how these affect the susceptibility to distractions either in particular situations or in relation to employing different strategies. This requires experimental research. Finally, our sample is demographically diverse, but limited to participants from Germany. Future research is needed to explore social media distraction in different cultural settings.

## Conclusion

Social media distractions can easily become a threat to task performance and well-being. For increasing users’ agency, future research should develop and test interventions that help users to reduce social media distractions. By identifying reasons for, situations of, and strategies against social media distraction, the present study provides an important step toward developing such interventions.

## Data Availability Statement

The datasets presented in this study can be found in online repositories. The names of the repository and accession number can be found below: OSF: https://osf.io/5pvj6/.

## Ethics Statement

The studies involving human participants were reviewed and approved by the Ethics Committee of the Department of Computer Science and Applied Cognitive Science, University of Duisburg-Essen, Duisburg, Germany. The participants provided their informed consent to participate in this study.

## Author Contributions

CK and OB designed the study. CK organized data collection, performed the statistical analysis, and wrote the first draft of the manuscript. CK and OB revised the manuscript and approved the submitted version.

## Conflict of Interest

The authors declare that the research was conducted in the absence of any commercial or financial relationships that could be construed as a potential conflict of interest.

## Publisher’s Note

All claims expressed in this article are solely those of the authors and do not necessarily represent those of their affiliated organizations, or those of the publisher, the editors and the reviewers. Any product that may be evaluated in this article, or claim that may be made by its manufacturer, is not guaranteed or endorsed by the publisher.

## Footnotes

1 We used a stratified sampling approach with specified subgroups regarding age (18–29; 30–39; 40–49; 50–59; and 60–69); at least 50 people were in each age group and there was a roughly equal gender distribution. We used this approach to ensure that the sample is demographically diverse and to avoid an unbalanced sample (e.g., younger persons might be more prone to participate in a study on social media).

2 In line with the recommendation by Stevens (2009), we collected data on more than 300 people, which allowed us to focus on factor loadings of 0.4 or greater in our EFA.

3 Measures are reported in order of appearance in the questionnaire. For exploratory purposes, we also assessed social media usage motivation and work-related self-efficacy. These are not further investigated here since a discussion exceeds the scope of this paper.

4 We used a broad definition of social media, including social networking sites and instant messaging apps.

5 We used an oblique rotation, since this is recommended when a correlation between factors is expected (Finch and French, 2015, p. 14) and considered reasonable (Stevens, 2009, p. 331). We assumed that the factors represent faces of distraction rather than clearly distinguishable dimensions and, hence, expected them to be correlated.

6 We used in R the “fa” function from “psych” package.

7 We excluded the item “I have nothing to do anyways” which had emerged from the interviews because we considered it to be conceptually different from distraction.
